# Radiosynthesis of a novel antisense imaging probe targeting LncRNA HOTAIR in malignant glioma

**DOI:** 10.1186/s12885-022-09170-7

**Published:** 2022-01-18

**Authors:** Jiongyu Ren, Xiyuan Zhang, Jiang Cao, Jiali Tian, Jin Luo, Yaping Yu, Fengkui Wang, Qian Zhao

**Affiliations:** 1 Department of Nuclear Medicine, General Hospital of Ningxia Medical University, Yinchuan, 750004 China; 2 Graduate School of Ningxia Medical University, Yinchuan, 750004 China; 3 XiangYa School of Medicine, CSU, Changsha, 410006 China

**Keywords:** LncRNA HOTAIR, Antisense oligonucleotide probe, Malignant glioma, SPECT imaging

## Abstract

**Background:**

Long non-coding RNA (LncRNA) HOTAIR was amplified and overexpressed in many human carcinomas, which could serve as a useful target for cancer early detection and treatment. The ^99m^Tc radiolabeled antisense oligonucleotides (ASON) could visualize the expression of HOTAIR and provide a diagnostic value for malignant tumors. The aim of this study was to evaluate whether liposome-coated antisense oligonucleotide probe ^99m^Tc-HYNIC-ASON targeting HOTAIR can be used in in vivo imaging of HOTAIR in malignant glioma xenografts.

**Methods:**

The ASON targeting LncRNA HOTAIR as well as mismatched ASON (ASONM) were designed and modified. The radiolabeling of ^99m^Tc with two probes were via the conjugation of bifunctional chelator HYNIC. Then probes were purified by Sephadex G25 and tested for their radiolabeling efficiency and purity, as well as stability by ITLC (Instant thin-layer chromatography) and gel electrophoresis. Then the radiolabeled probes were transfected with lipofectamine 2000 for cellular uptake test and the next experimental use. Furthermore, biodistribution study and SPECT imaging were performed at different times after liposome-coated ^99m^Tc-HYNIC-ASON/ASONM were intravenously injected in glioma tumor-bearing mice models. All data were analyzed by statistical software.

**Results:**

The labeling efficiencies of ^99m^Tc-HYNIC-ASON and ^99m^Tc-HYNIC-ASONM measured by ITLC were (91 ± 1.5) % and (90 ± 0.6) %, respectively, and both radiochemical purities were more than 89%. Two probes showed good stability within 12 h. Gel electrophoresis confirmed that the oligomers were successfully radiolabeled no significant degradation were found. Biodistribution study demonstrated that liposome-coated antisense probes were excreted mainly through the kidney and bladder and has higher uptake in the tumor. Meanwhile, the tumor was clearly shown after injection of liposome coated ^99m^Tc-HYNIC-ASON, and its T/M ratio was higher than that in the non-transfection group and mismatched group. No tumor was seen in mismatched and blocking group.

**Conclusion:**

The liposome encapsulated ^99m^Tc-HYNIC-ASON probe can be used in the in vivo, real-time imaging of LncRNA HOTAIR expression in malignant glioma.

**Supplementary Information:**

The online version contains supplementary material available at 10.1186/s12885-022-09170-7.

Glioma is the most proliferative and reversible primary brain tumor in human beings [1]. Approximately 80% of the malignant tumors of the central nervous system are gliomas [2]. Although treatment approaches such as surgery, radiotherapy, and chemotherapy have been widely used, the median survival time of glioma patients is still limited to about 14 months [3, 4]. Therefore, new and more effective approaches are urgently needed. LncRNA, a kind of mRNA-like transcripts as 200 nt to 100 kilobases (kb), have no protein-coding potential, however its roles in gene transcription, translation, heredity and epigenetic regulation in tumors has gained increasing attention in recent years [4]. It was found that LncRNA is widely expressed in human cancers, indicating that it may be associated with cancer onset and progression [5]. Zhang et al. [6] identified 129 LncRNAs, which were at least 2-fold higher in gliomas than in normal brain tissue. Of these, HOTAIR were up-regulated with ascending malignancy grades, and its expression level in high-grade astrocytoma is significantly higher than that in normal brains, suggesting its potential roles in the occurrence and development of gliomas. HOTAIR, as the first LncRNA with trans-regulatory function, silencing tumor suppressor genes by mediates recruitment of polycomb repressive complex 2 (PRC2) [7, 8]. Previous literature reported that HOTAIR can affect the cell cycle, and the decrease of HOTAIR expression will lead to a significant increase in G0/G1 phase cells [3]. Meanwhile, HOTAIR is positively correlated with tumor cell proliferation, survival, invasion and resistance to treatment via molecules such as chromatin modifiers and ubiquitin ligases [9]. Previous studies have been found that the expression of HOTAIR is significantly higher in cancer tissues than in matched tumor-adjacent healthy tissues [9]. Thus, these studies suggested that HOTAIR is stable and measurable in body fluids, and the high expression of HOTAIR is a biomarker of tumor diagnosis, metastasis, drug resistance and poor prognosis. Therefore, it has diagnostic and prognostic value for gliomas [10, 11].

Antisense imaging is an attractive and non-invasive method to detect the expression of HOTAIR in gliomas. Because the antisense oligonucleotide sequence can combine with the target gene through base complementary pairing, we attach radionuclides to antisense oligonucleotides so that radiolabeled oligonucleotides can noninvasively detect the expression of HOTAIR with SPECT and directly express and quantify biological processes at the cellular or subcellular level [12, 13].

Antisense oligonucleotide (ASON) technique uses a completely (or nearly complete) complementary single strand of 15 to 25 nucleotides that binds to the target gene, which can lead to gene silencing or interfere with the processing or translation of RNA [14, 15]. In addition, preclinical studies have shown that after chemical synthesis and modification of ASON (Antisense oligonucleotide), the nuclease degradation rate is decreased, while enhanced the affinity of plasma binding protein, the tissue biological distribution is rapid, thus makes ASON a good biological effect and stability [16, 17], which can be used as a feasible strategy for glioma treatment and detection. Compared with MRI, CT and other traditional imaging techniques for the diagnosis of gliomas, antisense imaging detects the expression of specific genes rather than anatomical variation [13]. As a molecular imaging technology, it has attracted much attention because of its high sensitivity, high resolution, and short acquisition time [18]. At the same time, compared with surgery, antisense oligonucleotide probe is non-invasive and would reveal the molecular changes of glioma at relatively early stages.

Considering the role of HOTAIR in tumorigenesis and treatment, as well as the efficiency, specificity, and irreversibility of antisense oligonucleotides binding to the target, the objective of the present study was to explore the ^99m^Tc labeled antisense oligonucleotide probe targeting HOTAIR, evaluate its characteristics in vitro, and observe whether it can be used in the imaging of gliomas.

## Materials and methods

The 19-base oligonucleotide probes targeting LncRNA HOTAIR were chemically synthesized and modified from Shanghai Shangon Bioengineering Co., Ltd. The ASON (Antisense oligonucleotide) sequence was designed as 5′- AATTCTTAAATTGGGCTGG − 3′, which was completely complementary to the HOTAIR fragment, and the ASONM (mismatched antisense oligonucleotides) sequence was 5′-AATACTTAGATTAGGCAGG-3′ (The underlined part is the substituted nucleosides). Two probes were modified with 2′ methylation (2′-O-methyl) at both ends of the sequences, two bases at each end were phosphonothioate modified to improve their stability, NH_2_C_6_ is connected to 5′ end. Ultimately, the synthetic structure was 5′-NH_2_C_6_-ASON/ASONM-3′. ^99m^Tc is obtained from the ^99m^Tc radionuclide generator produced by China Atomic Energy Research Institute.

### Synthesis and labeling of the probe

0.2 mg ASON (Antisense oligonucleotide) dissolved in 50 μL buffer (2 mol/L NaCl, 0.5 mol/L NaHCO_3_, 2 mmol/L EDTA), 1 mg HYNIC (TriLink, US) dissolved in 100 μL DMF solution. Mixed them at a molar ratio of 25:1(HYNIC: ASON) and avoid light for 1 h. Then the mixture above were added with 60% methanol to the total volume of 500 μL, using an ultrafilter tube (Sartorius, GER) and centrifuged 10 min at 13000×g (ensure that the volume after centrifugation was less than 50 μL) to obtain HYNIC-ASON. Next, 100 μL Tricine(100 mg/mL), 20 μL ^99m^Tc(222 MBq) as well as 4 μL fresh SnCl_2_•2H_2_O (1 mg/mL) were added to HYNIC-ASON in turn and reacted for 60 min. After the reaction, using Sephadex G25(GE, US) to separate and purify. Fifteen tubes of eluates were collected, then the radioactivity counts and nucleic acid concentration of each tube were detected respectively. The tube with the highest radioactivity counts and nucleic acid concentration was taken for follow-up experiments. ASONM (mismatched ASON) probes were prepared by the same method.

### Serum stability

Fresh human serum was provided by volunteers in our department. All the volunteers obtained verbal consent. The study was approved by the review committee and the ethics committee of the General Hospital of Ningxia Medical University. ^99m^Tc-HYNIC-ASON was incubated in saline and fresh human serum at 37 °C and room temperature, respectively (the volume ratio of the probe to serum/saline was 1:1). The radiochemical purity was detected by ITLC (Instant thin-layer chromatography) at 0, 2, 4, 6, 8, 12 h.

### Agarose gel electrophoresis

To identify the integrity of ^99m^Tc-HYNIC-ASON and eliminate the degradation after labeling. 1% agarose gel was configured, followed by an unbonded ASON (Antisense oligonucleotide) sample, ^99m^Tc, ^99m^Tc-HYNIC-ASON before and ^99m^Tc-HYNIC-ASON after purification. The voltage was 120 V, electrophoresis for 20 min, then the band was observed under UV.

### Cell culture and transfection

U87 glioma cells were purchased from the Chinese Academy of Sciences, and cultured in DMEM (Invitrogen, US) medium containing 15% fetal bovine serum (ABW, CHN) and 1% antibiotics in a CO_2_ incubator at 37 °C for 24 h, then passaged when the cell density reached 90%.

Transfection for animal experiments (Lip-^99m^Tc-HYNIC-ASON and Lip-^99m^Tc-HYNIC-ASONM). Liquid A: 10 μg purified ^99m^Tc-HYNIC-ASON (or^99m^Tc-HYNIC-ASONM) were added to 300 μL DMEM without serum and antibiotics; Liquid B: 25 μL Lipofectamine 2000 (Invitrogen, US) were added to 275 μL DMEM, then liquid A and B were placed at room temperature for 5 min respectively, then mixed two of them for 20 min to complete transfection.

### Cellular uptake

U87 cells were inoculated in a 12-well plate at 5 × 10^5^ density and cultured overnight in DMEM containing 15% FBS without antibiotics. The cells were divided into liposome transfected and non-transfected groups. In the transfection group, a mixture containing 200 μL DMEM, 0.5 μg ^99m^Tc-HYNIC-ASON (or ^99m^Tc-HYNIC-ASONM) (37 kBq) and 3 μL Lipofectamine 2000 was added into each well; In the non-transfection group, 0.5 μg ^99m^Tc-HYNIC-ASON (or ^99m^Tc-HYNIC-ASONM) (37 kBq) in 200 μL DMEM was added directly into the medium. After cultured in the 37 °C incubator, the samples were collected at 0.5 h, 2 h, 4 h and 6 h, each well was washed three times with 100 μL PBS for 3 times, the counts of radioactive medium and PBS were defined as Cout. Then the cells were lysed with trypsin containing EDTA and washed for 3 times, the radioactivity counts of cells, lysis solution and PBS were defined as Cin. The radioactivity counts of Cin and Cout were measured by γ radioimmunoassay counter (Chinese Academy of Metrology), and the uptake rate of cells to the probe at each time was calculated. Calculation formula: cell uptake rate = Cin/ (Cin + Cout) × 100%.

### Animal xenograft model

BALB/c nu/nu mice (female, weight ± SD, 20 ± 6 g, age 3 ~ 4wk) were fed in the Experimental Animal Center of Ningxia Medical University. Malignant glioma U87 cells (5 × 10^11^) were subcutaneously injected into the right fore axilla of each mouse. When the tumor reached 1.5–2.0 cm, it was used in the follow-up experiment. All animal experiments have passed the ethical review of the Animal Experimental Center of Ningxia Medical University, and were conducted under the guidelines of the Animal Welfare Committee.

### Biodistribution studies

Twenty nude mice were randomly divided into 5 groups with 4 mice in each group. Lip-^99m^Tc-HYNIC-ASON (or Lip-^99m^Tc-HYNIC-ASONM) 1 μg, 2.59 MBq (100 μL) was injected into the tail vein. Then the mice were euthanized by cervical dislocation at 1 h, 2, h 3 h, 4 h and 6 h after 100 μL of blood was taken from the ophthalmic vein. After that, tissues like heart, liver, spleen, kidney, stomach, small intestine, bladder, muscle, bone, and tumor were removed and weighed, then radioactivity counts were measured. The distribution results were recorded as the percentage of radioactivity per gram of tissue (% ID/g).

### SPECT imaging

Images were performed by a SPECT scanner at 1 h, 2 h, 4 h, 6 h, and 8 h respectively. 4 μg, 14.8 MBq (150 μL) Lip-^99m^Tc-HYNIC-ASON (or Lip-^99m^Tc-HYNIC-ASONM) probe were injected into the tail vein. 4 μg, 14.8 MBq (150 μL) ^99m^Tc-HYNIC-ASON for non-transfected group. In the blocking group, 10 μg liposome-transfected ASON was injected 2 h in advance to block, and then Lip-^99m^Tc-HYNIC-ASON was injected. The collection counts were 100,000 and stored as a 64 × 64 matrix at 3.2 zooms, then ratio of T/M (Tumor/Muscle) and T/A (Tumor/Abdomen) were calculated over the regions of interest.

### Statistical analysis

All data are processed by SPSS22.0 statistical software, variables are represented by $\overline{\mathrm{X}}\pm \mathrm{SD}$. T test was used for comparison between the two groups. *P* < 0.05 is considered to be statistically significant.

## Results

### Synthesis and labeling of the probe

**Fig. 1:**
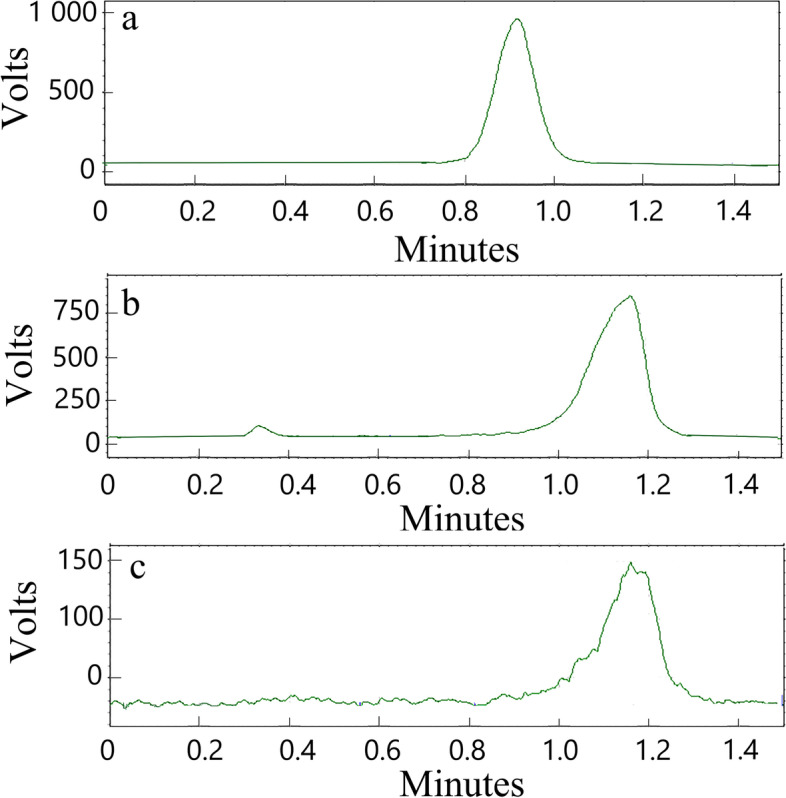
ITLC (Instant thin-layer chromatography) retention time of ^99m^Tc (**a**); ITLC retention time of ^99m^Tc-HYNIC-ASON before purification (**b**) and after purification (**c**). (ASON: Antisense oligonucleotide)

ITLC (Instant thin-layer chromatography) used saline as the developer, the radioactivity of the antisense probe before and after purification remained in the same position (1.18 Minutes) (Fig. 1). The labeling rate of ^99m^Tc-HYNIC-ASON and ^99m^Tc-HYNIC-ASONM measured by ITLC were (91 ± 1.5) % and (90 ± 0.6) %, respectively, and both radiochemical purities were more than 89% after purification.

### Radioactivity counts and nucleic acid concentration

**Fig. 2:**
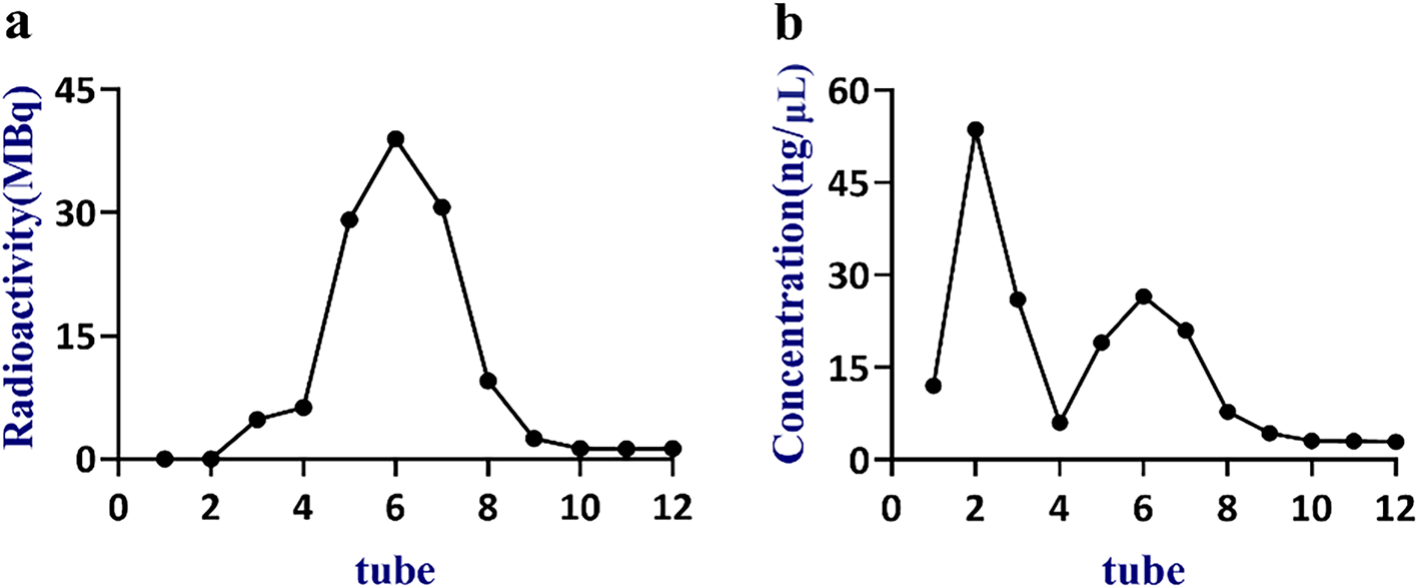
The radioactivity count (**a**) and nucleic acid concentration (**b**) of each tube of ^99m^Tc-HYNIC-ASON purified by Sephadex G25. (ASON: Antisense oligonucleotide)

In Fig. 2b, the unbonded ASON (Antisense oligonucleotide) fragments were filtered out by 1–4 tubes, and the eluent of 4–8 tubes were ^99m^Tc-HYNIC-ASON, which were coincided with the peak of radioactivity counts in Fig. 2a.

### Serum stability and agarose gel electrophoresis

**Fig. 3:**
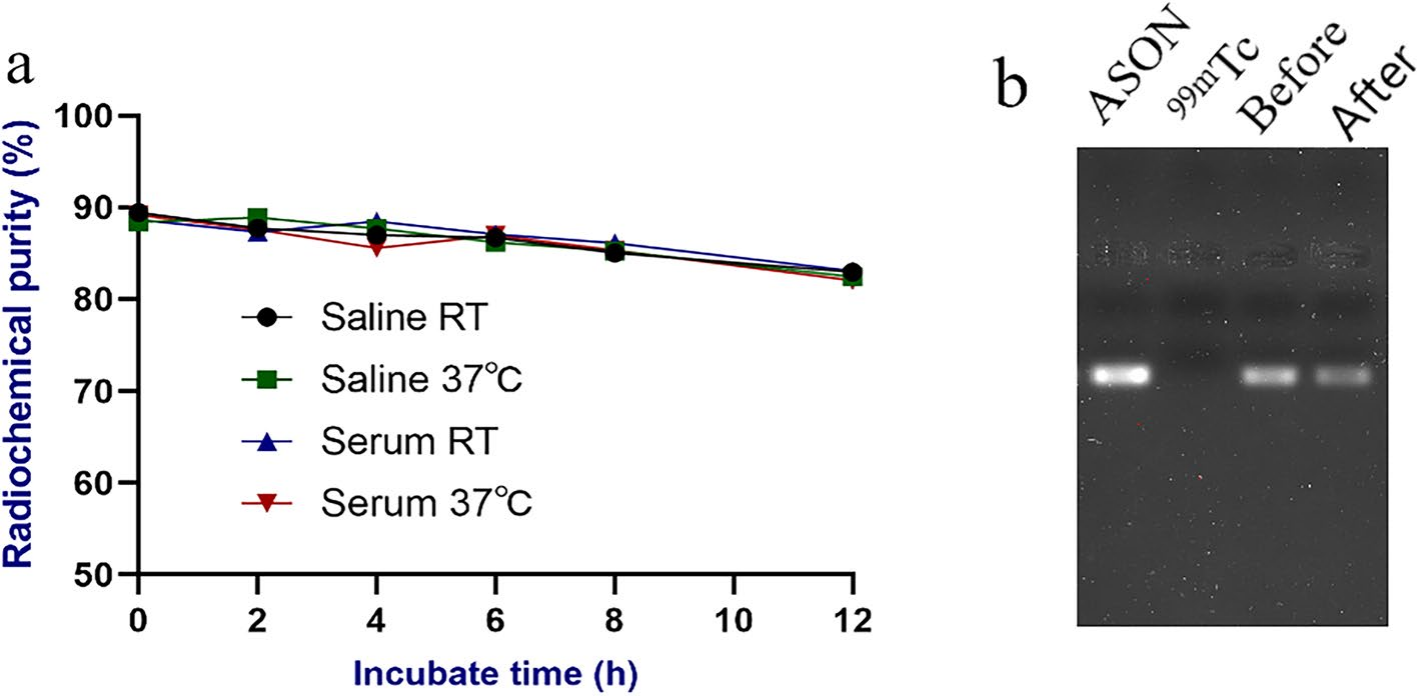
Probe stability identification. Radiochemical purity of ^99m^Tc-HYNIC-ASON incubated in saline and fresh serum during 6 h, at room temperature (RT) and 37 °C (**a**). The probe integrity was detected by agarose gel (from left to right: ASON sample, ^99m^Tc, ^99m^Tc-HYNIC-ASON before purification, ^99m^Tc-HYNIC-ASON after purification) (**b**). (full-length gel is presented in Supplementary Fig. 1.) (ASON: Antisense oligonucleotide)

The radiochemical purity of ^99m^Tc-HYNIC-ASON reached more than 80% in 12 h, and there was no significant difference between saline and fresh human serum (Fig. 3a). Figure 3b shows brighter bands before and after purification, indicating that the oligonucleotide probe has integrity and there is no obvious degradation, no band was seen in ^99m^Tc control group. The results showed that ^99m^Tc-HYNIC-ASON had good tolerance and stability in serum at 37 °C, which were similar to that in vivo.

### Cellular uptake

**Fig. 4:**
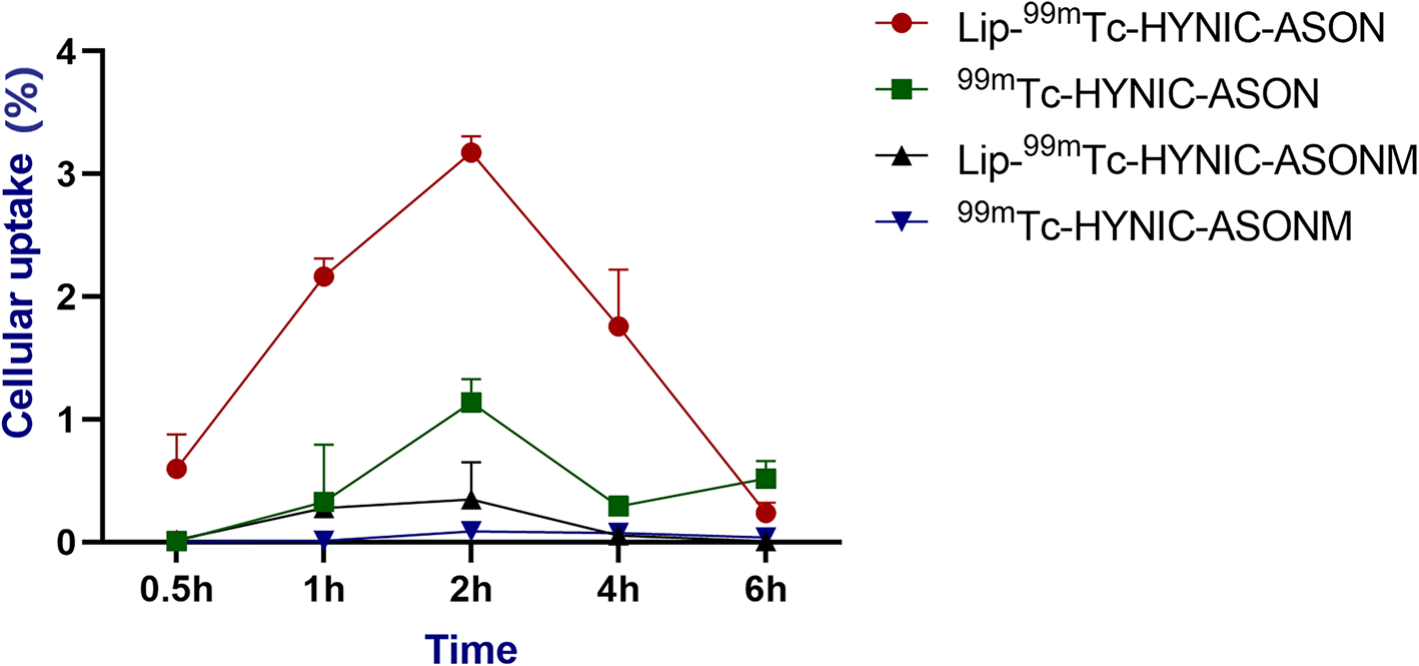
The in vitro cellular uptake rates of ^99m^Tc-HYNIC-ASON and mismatched probes with or without transfection at 30 min, 1 h, 2 h, 4 h and 6 h . (ASON: Antisense oligonucleotide)

The uptake rate of Lip-^99m^Tc-HYNIC-ASON in tumor cells reached the highest at 2 h (3.2%), while the uptake rate of non-transfection probes was only 1.2%. The results show that liposome can effectively carry the probe into the cell. Meanwhile, at each time point, the uptake rate of the transfection group was higher than that of the non-transfection group, and the highest cell uptake rate of Lip-^99m^Tc-HYNIC-ASONM was only 0.6%, indicating that the antisense oligonucleotide probe had high specificity, while the mismatched probe could not bind specifically. (*P* < 0.01) (Fig. 4).

### Biodistribution studies

Lip-^99m^Tc-HYNIC-ASON and mismatched probes had similar biological distributions except for the tumor radioactivity uptake. (listed in Tables 1 and 2). In the tumor, the concentration of mismatched probes was significantly lower than antisense probe concentration. At the same time, with the clearance of blood, the uptake of the two probes by the tumor decreased gradually. However, until 6 h after injection, the tumor uptake of Lip-^99m^Tc-HYNIC-ASON remained at a relatively high level.

**Table 1:** Biodistribution (%ID/g) of Lip-99mTc-HYNIC-ASON in Tumor-Bearing Mice

Tissue	1 h	2 h	3 h	4 h	6 h
**Heart**	**1.68 ± 0.38**	**1.73 ± 0.33**	**1.32 ± 0.1**	**0.82 ± 0.15**	**0.59 ± 0.21**
**Blood**	**1.37 ± 0.5**	**0.81 ± 0.58**	**0.56 ± 0.4**	**0.66 ± 0.17**	**0.18 ± 0.27**
**Liver**	**1.22 ± 0.42**	**1.28 ± 0.52**	**0.83 ± 0.32**	**0.88 ± 0.08**	**0.61 ± 0.29**
**Spleen**	**0.61 ± 0.05**	**0.45 ± 0.05**	**0.38 ± 0.14**	**0.29 ± 0.04**	**0.21 ± 0.04**
**Lung**	**1.5 ± 0.24**	**1.37 ± 0.35**	**1.01 ± 0.37**	**0.58 ± 0.08**	**0.37 ± 0.22**
**Kidney**	**6.25 ± 1.31**	**5.36 ± 1**	**5.38 ± 1.52**	**3.95 ± 0.67**	**2.63 ± 0.55**
**Stomach**	**2.3 ± 1.29**	**2.86 ± 0.46**	**2.7 ± 0.57**	**1.46 ± 0.14**	**1.31 ± 0.43**
**Small intestine**	**3.43 ± 0.82**	**3.17 ± 0.35**	**3.06 ± 0.82**	**2.21 ± 0.66**	**2.14 ± 0.67**
**bladder**	**11.89 ± 0.9**	**11.7 ± 0.75**	**9.81 ± 1.62**	**9.12 ± 1.66**	**8.75 ± 0.95**
**Skeletal muscle**	**0.81 ± 0.06**	**1.73 ± 0.23**	**2.68 ± 2.35**	**1.01 ± 0.48**	**1.76 ± 2.37**
**Bone**	**0.63 ± 0.32**	**1.96 ± 1.53**	**0.46 ± 0.32**	**0.87 ± 0.6**	**0.66 ± 0.6**
**Tumor**	**1.73 ± 0.13**	**1.22 ± 0.1**	**1.06 ± 0.22**	**0.82 ± 0.22**	**0.58 ± 0.1**

Each value represents average of 4 mice ± SD

**Table 2:** Biodistribution (%ID/g) of Lip-^99m^Tc-HYNIC-ASONM in Tumor-Bearing Mice

Tissue	2 h	4 h
**Heart**	**0.31 ± 0.13**	**0.35 ± 0.16**
**Blood**	**0.03 ± 0.01**	**0.06 ± 0.04**
**Liver**	**0.52 ± 0.25**	**0.6 ± 0.13**
**Spleen**	**0.19 ± 0.1**	**0.31 ± 0.07**
**Lung**	**0.43 ± 0.38**	**0.54 ± 0.14**
**Kidney**	**3.21 ± 0.54**	**2.73 ± 0.48**
**Stomach**	**0.42 ± 0.18**	**0.64 ± 0.19**
**Small intestine**	**0.21 ± 0.1**	**0.22 ± 0.13**
**bladder**	**2.62 ± 3.82**	**1.26 ± 0.77**
**Skeletal muscle**	**0.2 ± 0.13**	**0.45 ± 0.13**
**Bone**	**0.31 ± 0.14**	**0.3 ± 0.19**
**Tumor**	**0.43 ± 0.28**	**0.3 ± 0.1**

Each value represents average of 4 mice ± SD

**Fig. 5:**
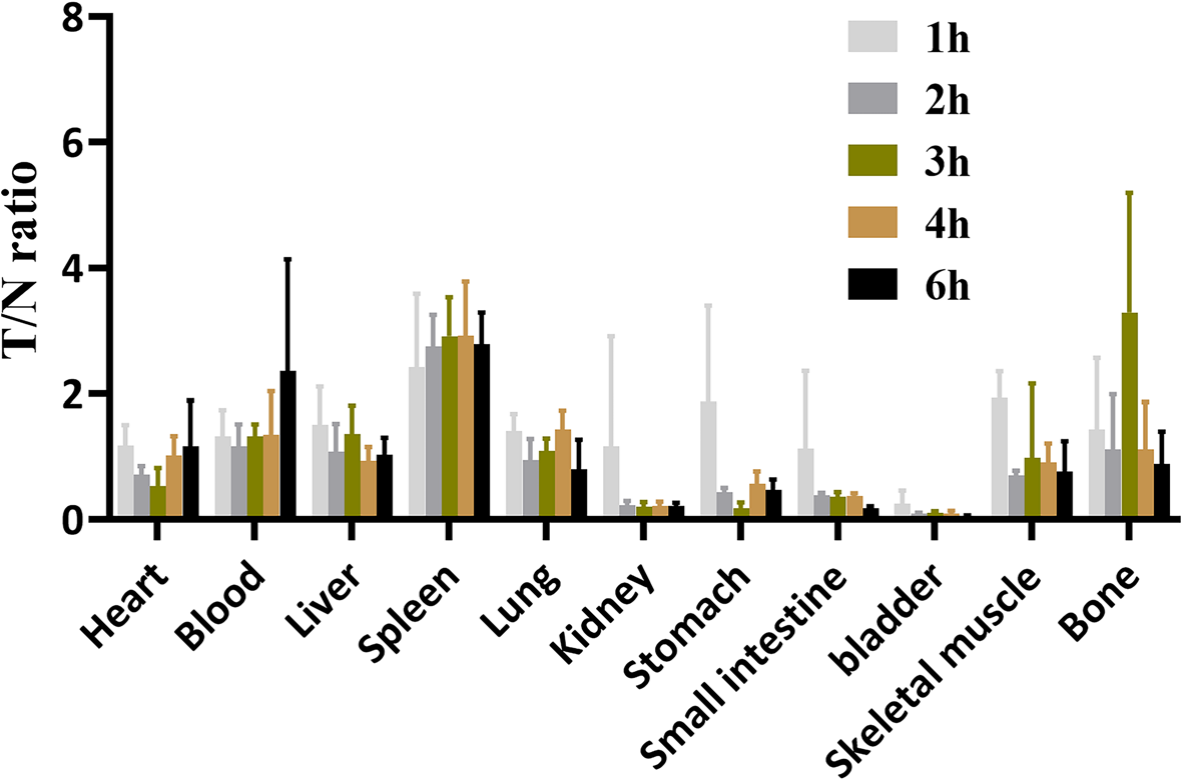
T/NT (Tumor/Non tumor tissues) ratio of Lip-^99m^Tc-HYNIC-ASON in different tissues of glioma nude bearing mice xenografts at 1 h, 2 h, 3 h, 4 h, and 6 h after injection. (ASON: Antisense oligonucleotide)

The two probes were mainly concentrated from the kidney and bladder, followed by the stomach and small intestine, indicating that the probes were cleared through the urinary and digestive systems. Secondly, for the organs with rich blood supply, such as the heart, liver, spleen, and lung, the uptake of the two probes decreased gradually due to the effect of blood clearance. Skeletal muscle and bone showed lower uptake of the probes. With the extension of time, after injection of Lip-^99m^Tc-HYNIC-ASON, the radioactivity in the kidney decreased rapidly, from (6.25 ± 1.31) % to (2.63 ± 0.55) %, while the radioactivity in the tumor decreased slowly in 6 h, from (1.73 ± 0.13) % to (0.58 ± 0.1) %. The results suggested that the Lip-^99m^Tc-HYNIC-ASON can be specifically aggregated in the tumor, which were also confirmed by the ratio of tumor to non-tumor (T/NT) of Lip-^99m^Tc-HYNIC-ASON (Fig. 5).

### SPECT imaging

**Fig. 6:**
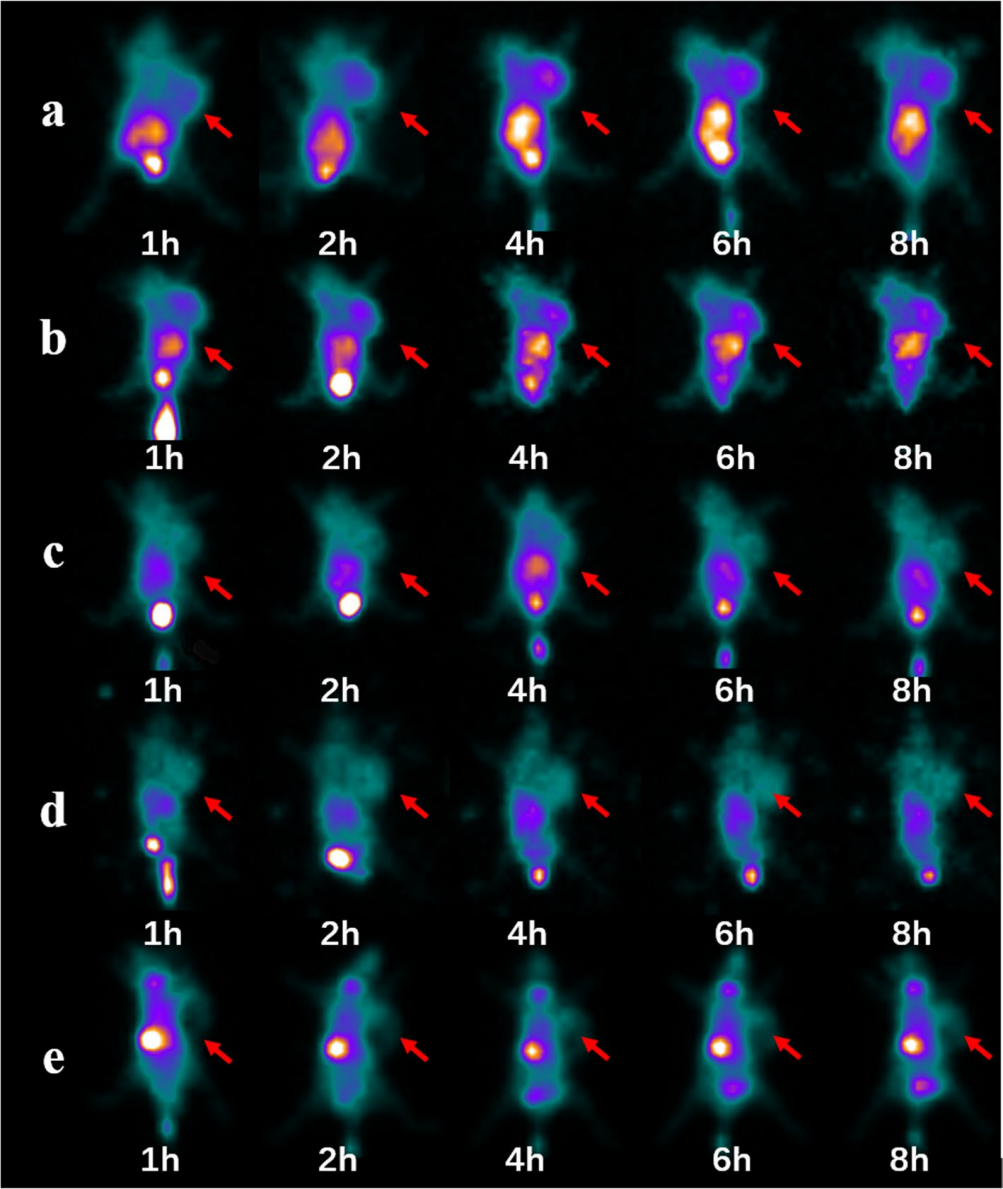
SPECT Imaging at 1 h, 2 h, 4 h, 6 h and 8 h after injected to glioma nude bearing mice models. **a**: Lip-99mTc-HYNIC-ASON; **b**: 99mTc-HYNIC-ASON **c**: Lip-99mTc-HYNIC-ASONM; **d**: Blocked; **e**: Tc-control. Tumor (arrow) was clearly visualized after injection of Lip-99mTc-HYNIC-ASON, meanwhile, the tumor could seen after injection of 99mTc-HYNIC-ASON, while tumor were not imaged at any time in mismatched、Blocked as well as Tc-control group. (ASON: Antisense oligonucleotide)

**Fig. 7:**
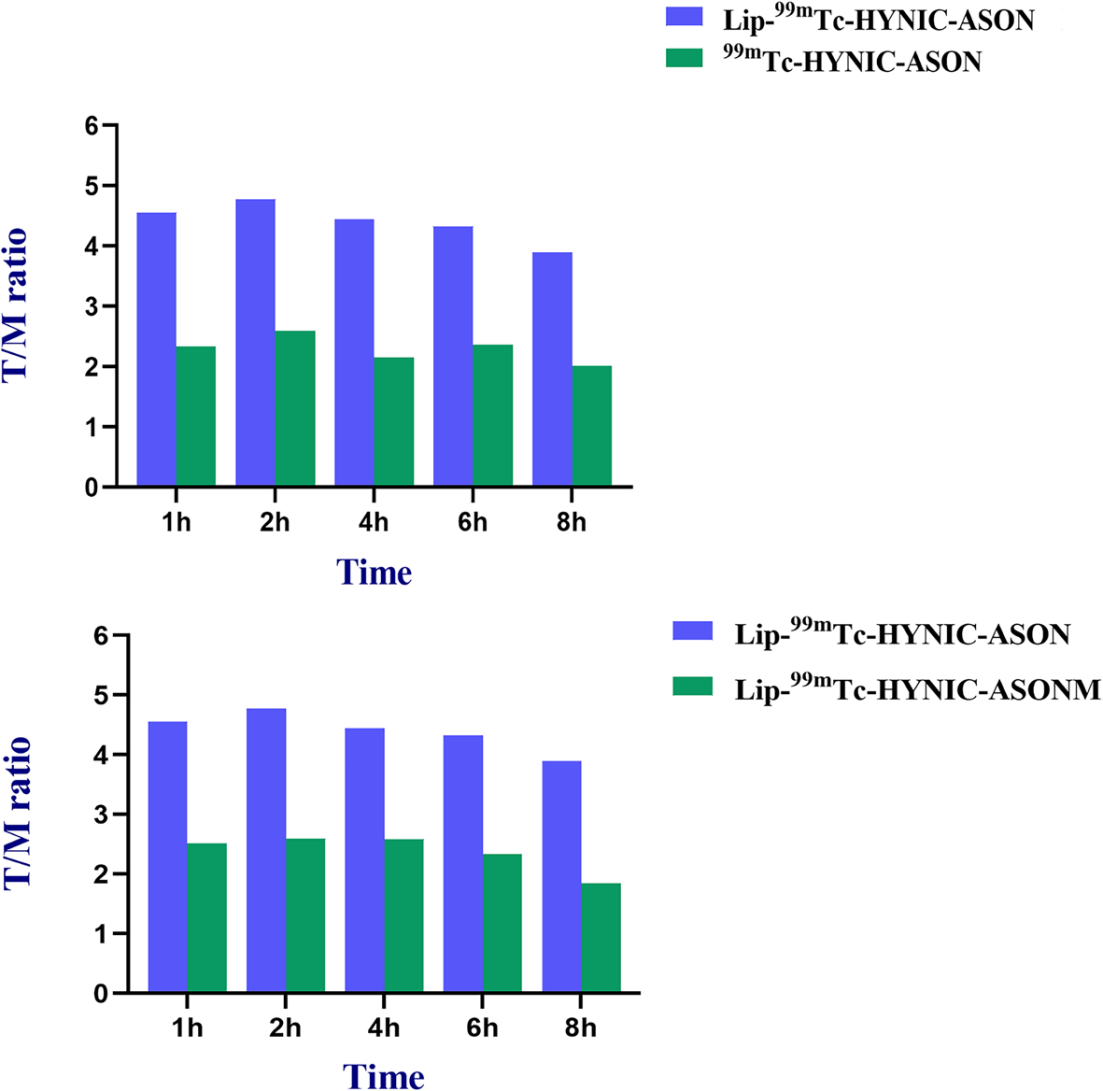
T/M (Tumor/Muscle) ratio of antisense probes with or without tranfection; T/M (Tumor/Muscle) ratio of liposome transfected antisense probes and mismatched probes. At all time, ratio of T/M of Lip-99mTc-HYNIC-ASON is significantly higher than non-transfection groups and mismatched groups. (ASON: Antisense oligonucleotide)

The static images were obtained within 8 h after injection. The tumor could be clearly shown after injection of Lip-^99m^Tc-HYNIC- ASON, and the best acquisition time was at 2 h.In contrast, the tumor was not shown within 8 hours in mismatched and blocking group (Fig. 6). At the same time, we injected the non-transfected probe for comparison, the tumor was shown within 8 h, however the T/M (Tumor/Muscle) ratio in non-transfected group (2.5%) was significantly lower than that in the liposome transfected group (4.8%) (Fig. 7), which was consistent with the results of cellular uptake experiment. And mismatched group also had a lower T/M (Tumor/Muscle) ratio when compared with liposome transfected group.

## Discussion

With the successful application of antisense imaging combined with radionuclide tracer technique, precise tracking method at the molecular level is used for tumor localization and therapy. In the present study, we successfully radiolabeled antisense oligonucleotide probe targeting LncRNA HOTAIR to track the expression of HOTAIR in glioma cells. We found that, in the labeling experiment, the chemically synthesized probes can successfully connect with ^99m^Tc and obtain a higher labeling rate; At the cellular level, liposomes can effectively carry the probes to tumor cells; From imaging results, the tumors were clearly imaged within 8 h after injection of antisense probe. While, the tumors were not apparent at any time after injection of the mismatched probe.

In this study, we changed the ratio of HYNIC to ASON (25:1) and replaced the buffer solution (2 mol/L NaCl, 0.5 mol/L NaHCO_3_, 2 mmol/L EDTA) that dissolved ASON (Antisense oligonucleotide) to improve the labeling rate. The effect is obvious. The labeling rate of the antisense probe and mismatched probe were both more than 90%, but the radiochemical purity is lower than that of other literature [19, 20].

One of the main factors affecting the binding of antisense probe is the low uptake rate of antisense oligonucleotide (ASON) probe in tumor cells [21]. Thus, we chose liposome as the carrier. As a cationic carrier, liposomes bind to anionic oligomers by simple charge attraction, which makes lipophilic oligonucleotides easily pass through the cell membrane. Therefore, the increase of antisense oligonucleotides can be observed in cells [22].

In this study, we compared liposome-transfected and non-transfected antisense probes, the results showed that the highest uptake rate of Lip-^99m^Tc-HYNIC-ASON in tumor cells was 3.2%, while that of non-transfected probes was only 1.2%. It shows that the liposome can effectively carry the probe into the cell. At the same time, the transfer of liposomes on the cell membrane is not a one-way street, because the mismatched probes encapsulated by liposomes do not bind specifically and do not accumulate a large amount of radioactivity in the cells can explain this problem. According to the biological distribution study, Lip-^99m^Tc-HYNIC- ASON has clearly accumulated in the tumor, meanwhile, the most radioactivity is accumulated in the kidney and bladder, indicating that the probe is mainly excreted through the urinary and digestive system. However, there is not much radioactivity accumulation in the liver, which is different from other reports that the liver has the highest radioactivity uptake in all organs [12, 13, 20, 23]. The reason might be that the molecular of Lip-^99m^Tc-HYNIC-ASON probe is small and does not need to be digested by macrophages when passing through the liver, hence, the radiolabeled antisense probe we developed caused a short retention time and mild potential toxicity to the liver.

In the imaging experiment, in the antisense group of liposome transfection, the tumor was clearly visible, and there was a lot of radioactivity in the abdomen and bladder, which was consistent with the biological distribution. No tumor was found in the blocking group within 8 h, indicating that this antisense oligonucleotide sequence targeting HOTAIR is highly specific, which can block the binding of ^99m^Tc-HYNIC-ASON to HOTAIR. The fact that there is no imaging in the mismatch group also shows the specificity of the antisense probe. Secondly, although the non-transfected antisense probe is also clustered in the tumor site, the T/M ratio of the non-transfected antisense probe is significantly lower than that of the transfected antisense probe, indicating the importance of liposomes in imaging.

In summary, these results demonstrated that ^99m^Tc-HYNIC-ASON can be successfully synthesized and used for glioma-specific imaging via transfected with lipofectamine. However, there are still some deficiencies and problems in this study that need to be addressed in future investigations. First of all, by changing the volume of the reaction solution, the concentration of SnCl_2_ ·2H_2_O as well as the reaction time, the radiochemical purity of the probes was still lower than that of other experiments (more than 98%). We will make further experiments, such as changing the reaction temperature and reaction concentration to improve the radiochemical purity. Secondly, the high uptake of radioactive probes by the bladder and kidney will blur the display of the tumor and affect the image quality. Furosemide, as a highly effective diuretic, has a strong diuretic effect and can quickly and briefly dilate renal blood vessels. Therefore, the application of furosemide may help to accelerate radioactive excretion and make the tumor develop more clearly. Third, high uptake of stomach and small intestine can cause radiation damage to tissue and lead to dysfunction. We speculate that the use of paroles is helpful to reduce high uptake and mucosal radiation damage.

## Conclusion

The liposome coated ^99m^Tc-HYNIC-ASON probe can be used for real-time imaging of LncRNA HOTAIR expression in malignant gliomas in vivo. The probe has good stability and targeting ability, which is a new type of non-invasive probe.

## Supplementary Information

**Additional file 1 **: **Supplementary Fig 1**. Full-length of gel.

## Data Availability

All data generated or analyzed during this study are included in this published article [and its supplementary information files]**.**

## Abbreviations

LncRNA: Long non-coding RNA
ASON: Antisense oligonucleotide
ASONM: Mismatched antisense oligonucleotide
ITLC: Instant thin-layer chromatography
T/NT ratio: Tumor / non-tumor ratio
T/M ratio: Tumor /muscle ratio
T/A ratio: Tumor /Abdomen ratio
