# Supplementary material for: Radiosynthesis of a novel antisense imaging probe targeting LncRNA HOTAIR in malignant glioma
Source: BMC Cancer. 2022 Jan 18;22:79. doi: 10.1186/s12885-022-09170-7 (PMC8767688; doi:10.1186/s12885-022-09170-7)
Supplement: Supplementary file 1 — Additional file 1 : Supplementary Fig 1. Full-length of gel. [file 12885_2022_9170_MOESM1_ESM.docx]

**Radiosynthesis of a novel antisense imaging probe targeting LncRNA HOTAIR in malignant glioma**

Jiongyu Ren ^1,2^, Xiyuan Zhang^3^, Jiang Cao ^1,2^, Jiali Tian^1,2^, Jin Luo ^1,2^, Yaping Yu ^1,2^, Fengkui Wang ^1^, Qian Zhao ^1*^

1. General Hospital of Ningxia Medical University, Yinchuan 750004 China
2. Graduate School of Ningxia Medical University, Yinchuan 750004 China

3. XiangYa School of Medicine, CSU, Changsha, 410006, China

Jiongyu Ren Email：[542492540@qq.com](mailto:542492540@qq.com)

Xiyuan Zhang Email：15378998097@163.com

Jiang Cao Email：517009445@qq.com

Jiali Tian Email：1830214025@qq.com

Jin Luo Email：2227434054@qq.com

Yaping Yu Email：2294440629@qq.com

Fengkui Wang Email：fery201314@163.com

**Corresponding author：**Qian Zhao, Department of Nuclear Medicine, General Hospital of Ningxia Medical University, Yinchuan 750004 China.

E-mail: [cecilia_hh@126.com](mailto:cecilia_hh@126.com) Tel:13895675766

**Supplementary information**


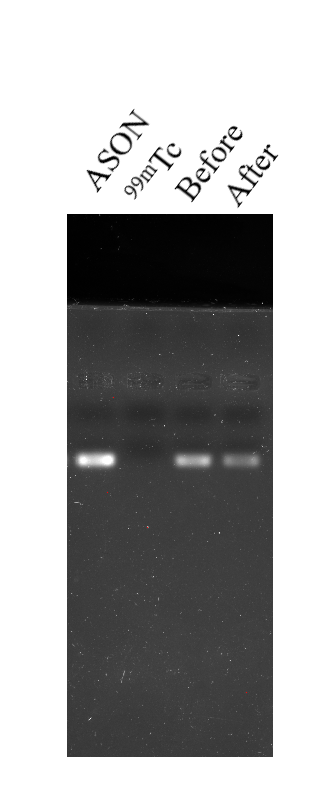


**Supplementary Fig 1: full-length of gel**
